# Association between circulating leukocyte subtype counts and carotid intima-media thickness in Japanese subjects with type 2 diabetes

**DOI:** 10.1186/1475-2840-12-177

**Published:** 2013-12-27

**Authors:** Takeshi Matsumura, Kayo Taketa, Hiroyuki Motoshima, Takafumi Senokuchi, Norio Ishii, Hiroyuki Kinoshita, Kazuki Fukuda, Sarie Yamada, Daisuke Kukidome, Tatsuya Kondo, Aya Hisada, Takahiko Katoh, Seiya Shimoda, Takeshi Nishikawa, Eiichi Araki

**Affiliations:** 1 Department of Metabolic Medicine, Kumamoto University, Kumamoto, Japan; 2 Department of Public Health, Faculty of Life Sciences, Kumamoto University, Kumamoto, Japan

**Keywords:** Leukocyte subtype counts, Carotid intima-media thickness, Diabetic macrovascular complication, Type 2 diabetes

## Abstract

**Background:**

An increased leukocyte count is an independent risk factor for cardiovascular events, but the association between leukocyte subtype counts and carotid atherosclerosis in patients with diabetes has not been determined. We therefore investigated the correlation between leukocyte subtype counts and intima-media thickness of the common carotid artery (CCA-IMT) in subjects with type 2 diabetes.

**Methods:**

This cross-sectional study involved 484 in-patients with type 2 diabetes (282 males and 202 females), who were hospitalized for glycemic control and underwent carotid ultrasonography at Kumamoto University Hospital between 2005 and 2011. Mean and maximum CCA-IMT was measured by high-resolution B-mode ultrasonography.

**Results:**

Univariate analyses revealed that mean CCA-IMT was positively correlated with age, systolic blood pressure, brachial-ankle pulse wave velocity (PWV), urinary albumin excretion and duration of diabetes, but was negatively correlated with diastolic blood pressure and fasting plasma glucose. Maximum CCA-IMT was positively and negatively correlated with the same factors as mean CCA-IMT except for fasting plasma glucose. Mean CCA-IMT was positively correlated with total leukocyte (*r* = 0.124, p = 0.007), monocyte (*r* = 0.373, p < 0.001), neutrophil (*r* = 0.139, p = 0.002) and eosinophil (*r* = 0.107, p = 0.019) counts. Maximum CCA-IMT was positively correlated with total leukocyte (*r* = 0.154, p < 0.001), monocyte (*r* = 0.398, p < 0.001), neutrophil (*r* = 0.152, p < 0.001) and basophil counts (*r* = 0.102, p = 0.027). Multiple regression analyses showed that monocyte count, age and PWV were significant and independent factors associated with mean CCA-IMT (adjusted R^2^ = 0.239, p < 0.001), and that monocyte count, age and urinary albumin excretion were significant and independent factors associated with maximum CCA-IMT (adjusted R^2^ = 0.277, p < 0.001).

**Conclusions:**

Monocyte counts were positively correlated with both mean CCA-IMT and maximum CCA-IMT in patients with type 2 diabetes. Monocyte count may be a useful predictor of macrovascular complications in patients with type 2 diabetes.

**Trial registration:**

Trial registry no: UMIN000003526.

## Background

Type 2 diabetes mellitus is associated with a high risk of cardiovascular diseases (CVD), and many patients with diabetes die from CVD, mainly caused by markedly advanced atherosclerosis [1]. Measurement of the intima-media thickness of the common carotid artery (CCA-IMT) by B-mode ultrasound was found suitable for monitoring the early stages of atherosclerosis [2]. Moreover, CCA-IMT has been reported to be an indicator of CVD [3,4]. On the other hand, increased CCA-IMT has also been observed in patients with type 2 diabetes [5-7] or metabolic syndrome [8], and asymptomatic hyperglycemic subjects were shown to have significantly higher CCA-IMT than healthy control subjects [9]. Therefore, CCA-IMT has been used as a marker of atherosclerosis progression in patients with type 2 diabetes.

Atherosclerosis is a chronic inflammatory process characterized by early leukocyte recruitment and progression to atherosclerotic plaque maturation [10]. Several epidemiologic studies have reported that an increased leukocyte count is a strong and independent risk factor for cardiovascular events [11-17] and for the prevalence and progression of subclinical carotid atherosclerosis [18-23]. However, Kuo et al. reported no association between total leukocyte count and CCA-IMT in asymptomatic subjects with abnormal complete blood cells in Taiwan [24]. On the other hand, the associations between leukocyte subtype counts and carotid atherosclerosis in patients with diabetes are largely unknown. Thus, this study was designed to investigate the associations between leukocyte subtype counts and carotid intra-media thickness in Japanese subjects with type 2 diabetes.

## Methods

### Study population

This cross-sectional study recruited 562 patients with type 2 diabetes who were hospitalized for glycemic control and underwent carotid ultrasonography at Kumamoto University Hospital between 2005 and 2011. Type 2 diabetes was diagnosed based on World Health Organization criteria [25]. Patients with type 1 diabetes were excluded, as were patients positive for glutamic acid decarboxylase antibody, those with a history of ketoacidosis, and patients dependent on insulin therapy for survival. Patients with severe hepatic disease, malignancy, or acute/chronic inflammatory disease were also excluded. A total of 484 subjects with type 2 diabetes (282 men, and 202 women) were analyzed. Patient characteristics are listed in Table 1. All subjects included in this study were Japanese. Each patient participated in a detailed interview of his/her personal disease and smoking history. Information on smoking habits was assessed by a standardized questionnaire. Smoking status of patients was classified as never having smoked, former smoker (ceased smoking for at least 1 year), or current smoker. In this study, former and current smokers were pooled and compared with never smokers.

**Table 1:** **Subject characteristics (*n*** **= 484)**

	
Age (years)	59.7 ± 12.6
Sex (% male)	58.3
Duration of diabetes (years)	13.2 ± 10.2
BMI (kg/m^2^)	26.1 ± 11.4
SBP (mmHg)	129.7 ± 20.5
DBP (mmHg)	75.5 ± 12.7
FPG (mmol/l)	9.0 ± 3.2
HbA1c (%)	9.1 ± 2.1
TC (mmol/l)	5.21 ± 1.30
TG (mmol/l)	1.91 ± 1.50
HDL cholesterol (mmol/l)	1.28 ± 0.38
LDL cholesterol (mmol/l)	3.06 ± 1.05
non-HDL cholesterol (mmol/l)	3.93 ± 1.30
hs-CRP (mg/l)	0.22 ± 0.41
mean-CCA-IMT (mm)	0.97 ± 0.38
max-CCA-IMT (mm)	1.11 ± 0.53
baPWV (cm/s)	1748.4 ± 360.0
UAE (mg/day)	363.1 ± 891.6
total leukocyte count (/mm^3^)	6258.1 ± 1656.8
Monocyte count (/mm^3^)	340.5 ± 106.5
Neutrophil count (/mm^3^)	3587.2 ± 1232.6
Lymphocyte count (/mm^3^)	2079.5 ± 680.4
Eosinophil count (/mm^3^)	208.1 ± 145.5
Basophil count (/mm^3^)	32.2 ± 25.9
Current or former smokers (%)	52.5
Hypertension (%)	61.8
Hyperlipidemia (%)	55.8
Diabetic microangiopathy (%)	
Retinopathy	56.6
Neuropathy	49.8
Nephropathy	47.3
Cardiovascular disease (%)	18.8
Diabetes medication (%)	
Oral hypoglycemic agents	50.0
Insulin	28.3
Oral hypoglycemic agents + insulin	7.6
Statins (%)	22.3
ARBs and/or ACEIs (%)	29.8
CCBs (%)	29.5
Ant-platelet agents (%)	17.1

Data are means ± SD.

SBP, systolic blood pressure; DBP, diastolic blood pressure; FPG, fasting plasma glucose; TC, total cholesterol; TG, triglyceride; hs-CRP, high sensitive C-reactive protein; CCA-IMT, intima-media thickness of common carotid artery; baPWV, brachial ankle pulse wave velocity; UAE, urinary albumin excretion; ARBs, angiotensin II receptor blockers; ACEIs, angiotensin converting enzymes; CCBs, calcium channel blockers.

Hypertension was defined by a blood pressure > 130/80 mmHg or treatment with antihypertensive agents. Hyperlipidemia was defined as TC > 5.7 mmol/l and/or TG > 1.7 mmol/l or treatment with antihyperlipidemic agents. Cardiovascular disease was defined as stroke, ischemic heart disease and arteriosclerosis obliterans.

The study protocol was approved by the Human Ethics Review Committee of Kumamoto University (Protocol Number: 1171), and all subjects provided written informed consent.

### Laboratory measurements

Within 2 days of admission, blood samples were collected from all participants in the early morning after an overnight fast. An automated hematologic analyzer (XE-2100, Sysmex, Kobe, Japan) was used to measure total and differential leukocyte counts. Fasting plasma glucose (FPG), high-sensitivity C-reactive protein (hs-CRP), glycosylated hemoglobin (HbA1c), serum total cholesterol (TC), triglycerides (TG) and high-density lipoprotein (HDL) cholesterol were measured by standard methods. Low-density lipoprotein (LDL) cholesterol was determined using the Friedewald formula [26]. Non-HDL cholesterol was calculated by subtracting HDL cholesterol from TC. HbA1c (%) was estimated as National Glycohemoglobin Standardization Program (NGSP) equivalent values (%), calculated using the formula HbA1c (%) = 1.02 × HbA1c [Japan Diabetes Society (JDS)] (%) + 0.25%, considering the relational expression of HbA1c (JDS) (%) measured by the previous Japanese standard substance and measurement methods and HbA1c (NGSP) [27]. Urinary albumin excretion (UAE) was assessed and performed in three consecutive 24 h urinary collections. For collection of the urine sample, a 3-liter plastic container was used, and the volume of total urine was measured. Albumin levels were determined by standard immunonephelometric assay. UAE was expressed as milligrams per 24 h of the mean of three measures.

### Carotid ultrasonography and brachial-ankle pulse wave velocity (baPWV) measurement

IMT was measured ultrasonographically using the Shimadzu SDU-2200 (Shimadzu Co., Ltd, Kyoto, Japan) at a transducer frequency of 5–10 MHz. This system provides an axial resolution of 0.30 mm. Intima Scope software (Media Cross Co. Ltd, Tokyo, Japan) was used for computer-assisted acquisition, processing, storage of B-mode images, and calculation of IMT. The near and far walls of the common carotid arteries, the carotid bifurcations, and the origins (first 2 cm) of the internal carotid arteries were scanned longitudinally and transversely to assess the occurrence of plaques. IMT was defined and measured as previously described [2]. We used the scans of the far wall CCAs for mean IMT, because several methodological reviews [28-30] have reported that i) IMT can only be measured accurately in the far wall position, and ii) multiple, good-quality scans may be obtained from nearly every patient from the CCAs, while the percentage of missing images from the internal carotid arteries is higher. The mean values of CCA-IMT (mean-CCA-IMT) and maximum values of CCA-IMT (max-CCA-IMT) were used in this study.

All carotid artery measurements were performed by three sonographers, who, at the time of examination, were unaware of detailed clinical information on each patient. Mean-CCA-IMT was measured using an automated edge-detection algorithm based on significant changes in density of a section between the lumen and subadventitial structures perpendicular to the vessel wall. The software estimated lines for the lumen-intima interface and the media-adventitia interface based on 30-point pixels per 3 mm from tertiary multiple regression analysis incorporating the least squares method, which was designed to increase accuracy and reproducibility while reducing variability for measurements of IMT. Using longitudinal views of both the right and left CCAs, two measurements were made at the 20 mm segment distal to the carotid bulbs. For each measurement the average values were calculated automatically. Sonographers repeated ultrasound examinations in 42 subjects to ensure the reproducibility of the measurements. The correlation coefficients for the intra- and interobserver variations were 0.91 (p < 0.001) and 0.89 (p < 0.001), respectively, for mean-CCA-IMT, and were 0.89 (p < 0.001) and 0.83 (p < 0.001), respectively, for max-CCA-IMT.

Systolic blood pressure (SBP) and diastolic blood pressure (DBP) were measured twice with the patient in a sitting position after a 5-min rest. Left and right baPWV were measured automatically using an ABI-form (BP-203RPE II; Nippon Colin, Komaki, Japan). The highest values of SBP, DBP and baPWV from the left and the right sides were used in this study. Regarding reproducibility, the coefficient of variation was less than 5% for baPWV [31].

### Statistical analysis

All statistical analyses were performed using SPSS software version 14 for Windows (SPSS Inc., Chicago, IL, USA). All data are given as means ± standard deviation or as actual numbers. Since some variables showed a skewed distribution (i.e., mean CCA-IMT, max-CCA-IMT, duration of diabetes, TG, hs-CRP, UAE, monocyte count, eosinophil count and basophil count), they were logarithmically transformed before the analysis. The influences of variables on several factors including mean-CCA-IMT, max-CCA-IMT, total leukocyte count, monocyte count and neutrophil count were explored by simple linear analysis, using Pearson’s method for continuous variables. An unpaired Student *t*-test was used for normally distributed variables. For the multivariate analysis, stepwise linear regression was performed. The criteria for entering or keeping the variable in the model were p < 0.05 and p < 0.1, respectively, in the univariate analysis. In the case of collinear and conceptually intertwined variables (such as TC and LDL cholesterol, or HbA1c and FPG), alternative models were constructed. Finally, factors included in the stepwise multiple regression models for mean-CCA-IMT were age, sex, duration of diabetes, body mass index (BMI), SBP, FPG, baPWV, UAE, total leukocyte count, monocyte count, neutrophil count, eosinophil count and basophil count. Factors for max-CCA-IMT were age, sex, duration of diabetes, smoking status, BMI, SBP, baPWV, UAE, total leukocyte count, monocyte count, neutrophil count, eosinophil count and basophil count. A p value < 0.05 was considered statistically significant.

## Results

The characteristics of the study subjects are shown in Table 1. Mean age was 59.7 ± 12.6 years, and 58.3% of the subjects were male. The mean duration of diabetes was 13.2 ± 10.2 years, and the mean HbA1c was 9.1 ± 2.1%. Mean CCA-IMT, max-CCA-IMT and baPWV were 0.97 ± 0.38 mm, 1.11 ± 0.53 mm and 1748.4 ± 360.0 cm/s, respectively. The total leukocyte, monocyte, neutrophil, lymphocyte, eosinophil and basophil counts were 6258.1 ± 1656.8/mm^3^, 340.5 ± 106.5/mm^3^, 3587.2 ± 1232.6/mm^3^, 2079.5 ± 680.4/mm^3^, 208.1 ± 145.5/mm^3^ and 32.2 ± 25.9/mm^3^, respectively. Overall, 18.8% of the patients had a history of myocardial infarction and/or stroke.

**Figure 1:**
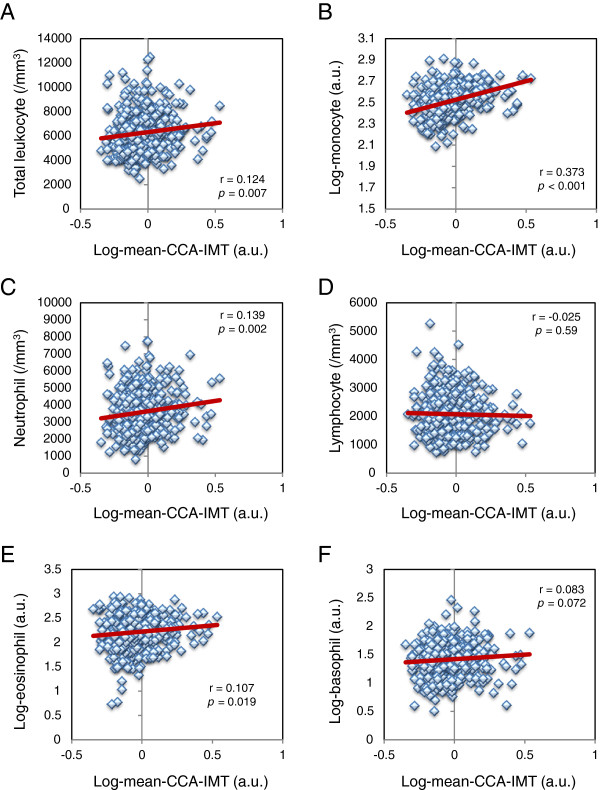
**Association of leukocyte subset counts with mean-CCA-IMT in patients with type 2 diabetes. A**, total leukocyte count; **B**, monocyte count; **C**, neutrophil count; **D**, lymphocyte count; **E**, eosinophil count; **F**, basophil count. Since mean-CCA-IMT, monocyte count, eosinophil count and basophil count showed skewed distribution, data were logarithmically transformed before the analysis. a.u., arbitrary unit.

**Figure 2:**
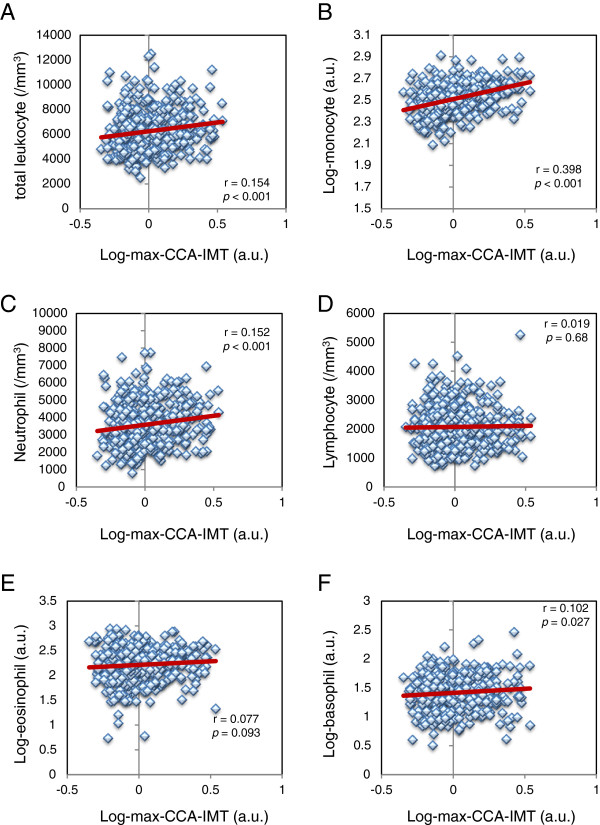
**Association of leukocyte subset counts with max-CCA-IMT in patients with type 2 diabetes. A**, total leukocyte count; **B**, monocyte count; **C**, neutrophil count; **D**, lymphocyte count; **E**, eosinophil count; **F**, basophil count. Since max-CCA-IMT, monocyte count, eosinophil count and basophil count showed skewed distribution, data were logarithmically transformed before the analysis. Units of leukocyte, neutrophil and lymphocytes were/mm^3^. a.u., arbitrary unit.

Univariate associations of mean-CCA-IMT and max-CCA-IMT with the clinical characteristics in the patients are shown in Table 2. Mean-CCA-IMT was positively correlated with age, duration of diabetes, BMI, baPWV, UAE and SBP, but negatively correlated with DBP and FPG (Table 2). Max-CCA-IMT showed similar correlations except for FPG (Table 2). Additional analyses were performed to determine the associations of leukocyte subset counts with mean-CCA-IMT or max-CCA-IMT in patients with type 2 diabetes (Table 2, Figures 1 and 2). Mean-CCA-IMT was positively correlated with total leukocyte, monocyte, neutrophil and eosinophil counts (Table 2). Max-CCA-IMT was positively correlated with total leukocyte, monocyte, neutrophil and basophil counts (Table 2). Both mean-CCA-IMT and max-CCA-IMT in males were significantly higher than in females (p = 0.015 and p = 0.006, respectively.). On the other hand, smoking was significantly associated with increased max-CCA-IMT (p = 0.007), but not with mean-CCA-IMT (p = 0.16).

**Table 2:** Correlation analysis of mean-CCA-IMT and max-CCA-IMT with metabolic parameters

	**mean-CCA-IMT**			**max-CCA-IMT**	
	*** r***	*** p***		*** r***	*** p***
Age	0.327	<0.001	Age	0.343	<0.001
Duration of diabetes	0.262	<0.001	Duration of diabetes	0.237	<0.001
BMI	−0.169	<0.001	BMI	−0.151	<0.001
FPG	−0.095	0.043	FPG	−0.044	0.35
HbA1c	−0.053	0.25	HbA1c	−0.011	0.81
TC	−0.038	0.42	TC	−0.038	0.41
TG	−0.010	0.83	TG	−0.022	0.64
HDL-C	−0.017	0.72	HDL-C	−0.021	0.65
LDL-C	−0.031	0.5	LDL-C	−0.016	0.74
non-HDL-C	−0.032	0.49	non-HDL-C	−0.032	0.49
hs-CRP	0.029	0.54	hs-CRP	0.000	0.99
max-CCA-IMT	0.798	<0.001	mean-CCA-IMT	0.798	<0.001
baPWV	0.292	<0.001	baPWV	0.286	<0.001
UAE	0.109	0.018	Urinary albumin	0.151	<0.001
SBP	0.145	0.002	SBP	0.135	0.003
DBP	−0.104	0.024	DBP	−0.092	0.045
Total leukocyte count	0.124	0.007	Total leukocyte count	0.154	<0.001
Monocyte count	0.373	<0.001	Monocyte count	0.398	<0.001
Neutrophil count	0.139	0.002	Neutrophil count	0.152	<0.001
Lymphocyte count	−0.025	0.59	Lymphocyte count	0.019	0.68
Eosinophil count	0.107	0.019	Eosinophil count	0.077	0.093
Basophil count	0.083	0.072	Basophil count	0.102	0.027

Data are means ± SD.

BMI, body mass index; SBP, systolic blood pressure; DBP, diastolic blood pressure; FPG, fasting plasma glucose; TC, total cholesterol; TG, triglyceride; HDL-C, high-density lipoprotein cholesterol; LDL-C, low-density lipoprotein cholesterol; hs-CRP, high sensitive C-reactive protein; CCA-IMT, intima-media thickness of common carotid artery; baPWV, brachial ankle pulse wave velocity; UAE, urinary albumin excretion. Since mean CCA-IMT, max-CCA-IMT, Duration of diabetes, TG, hs-CRP, UAE, Monocyte count, Eosinophil count and Basophil count showed skewed distribution, it was logarithmically transformed before the analysis.

We analyzed the univariate associations of total leukocyte, monocyte and neutrophil counts with the clinical characteristics of the patients with type 2 diabetes (Table 3). Total leukocyte count was positively correlated with BMI, SBP, DBP, TC, TG, LDL cholesterol, non-HDL cholesterol, hs-CRP, mean-CCA-IMT, max-CCA-IMT and UAE, but negatively correlated with age and HDL cholesterol (Table 3). The monocyte count was positively correlated with duration of diabetes, SBP, TC, TG, LDL cholesterol, hs-CRP, mean-CCA-IMT, max-CCA-IMT and UAE, and negatively correlated with HDL cholesterol (Table 3). The neutrophil count was positively correlated with BMI, SBP, DBP, TC, TG, non-HDL cholesterol, hs-CRP, mean-CCA-IMT, max-CCA-IMT and UAE, and negatively correlated with age and HDL cholesterol (Table 3). The monocyte count in males was significantly higher than that in females (p < 0.001). Smoking was significantly associated with an increased monocyte count (p < 0.001).

**Table 3:** Correlations between total leukocyte, monocyte and neutrophil counts with clinical characteristics

	**Total leukocyte count**		**Monocyte count**		**Neutrophil count**	
	***r***	***p***	***r***	***p***	***r***	***p***
Age	−0.197	<0.001	−0.007	0.884	−0.115	0.011
Duration of DM	0.006	0.89	0.102	0.025	0.031	0.5
BMI	0.219	<0.001	0.050	0.27	0.161	<0.001
SBP	0.127	0.005	0.126	0.006	0.184	<0.001
DBP	0.113	0.014	0.064	0.16	0.090	0.048
FPG	−0.024	0.61	−0.021	0.64	−0.046	0.33
HbA1c	0.083	0.07	0.057	0.21	−0.019	0.68
TC	0.146	0.001	0.036	0.44	0.096	0.038
TG	0.281	<0.001	0.125	0.007	0.163	<0.001
HDL-C	−0.171	<0.001	−0.113	0.014	−0.142	0.002
LDL-C	0.132	0.004	0.049	0.29	0.099	0.032
non-HDL-C	0.202	<0.001	0.073	0.12	0.142	0.002
hs-CRP	0.307	<0.001	0.215	<0.001	0.326	<0.001
mean-CCA-IMT	0.124	0.007	0.373	<0.001	0.139	0.002
max-CCA-IMT	0.154	<0.001	0.398	<0.001	0.152	<0.001
baPWV	−0.009	0.85	0.072	0.13	0.065	0.16
UAE	0.206	<0.001	0.124	0.007	0.243	<0.001

Data are means ± SD.

SBP, systolic blood pressure; DBP, diastolic blood pressure; FPG, fasting plasma glucose; TC, total cholesterol; TG, triglyceride; hs-CRP, high sensitive C-reactive protein; CCA-IMT, intima-media thickness of common carotid artery; baPWV, brachial ankle pulse wave velocity; UAE, urinary albumin excretion. Since mean-CCA-IMT, max-CCA-IMT, duration of diabetes, TG, hs-CRP, UAE, monocyte count, eosinophil count and basophil count showed skewed distribution, it was logarithmically transformed before the analysis.

We next performed stepwise multiple regression analysis to identify the independent factors associated with mean-CCA-IMT and max-CCA-IMT (Table 4). Monocyte count, age and baPWV were significant and independent factors associated with mean-CCA-IMT (adjusted R^2^ = 0.239, p < 0.001). On the other hand, monocyte count, age and UAE were significant and independent factors associated with max-CCA-IMT (adjusted R^2^ = 0.277, p < 0.001) (Table 4).

**Table 4:** Stepwise multiple regression analysis to identify factors associated with mean-CCA-IMT and max-CCA-IMT

	**Unstandardized**	**Standardized**	***p***
	**Regression coefficient**	**Regression coefficient**	
mean-CCA-IMT			
Monocyte count	0.3466	0.3260	<0.001
Age	0.0029	0.2675	<0.001
baPWV	0.0001	0.1446	0.004
max-CCA-IMT			
Monocyte count	0.4659	0.3448	<0.001
Age	0.0051	0.3674	<0.001
UAE	0.0357	0.1687	<0.001

Factors included in the stepwise multiple regression models for mean intima-media thickness of common carotid artery (mean-CCA-IMT) were age, gender, duration of diabetes, body mass index, systolic blood pressure, fasting plasma glucose, brachial ankle pulse wave velocity (baPWV), urinary albumin excretion (UAE), total leukocyte count, monocyte count, neutrophil count, eosinophil count and basophil count. Factors included in the stepwise multiple regression models for maximum CCA-IMT (max-CCA-IMT) were age, gender, duration of diabetes, smoking status, body mass index, systolic blood pressure, baPWV, UAE, total leukocyte count, monocyte count, neutrophil count, leosinophil count and basophil count. Since mean-CCA-IMT, max-CCA-IMT, duration of diabetes, TG, hs-CRP, UAE, monocyte count, eosinophil count and basophil count showed skewed distribution, it was logarithmically transformed before the analysis.

## Discussion

Several studies have reported that CCA-IMT is a useful marker for the progression of atherosclerosis in subjects with type 2 diabetes [3-5,32,33]. Here, we revealed that age, duration of diabetes, SBP and DBP were correlated with mean-CCA-IMT and max-CCA-IMT in Japanese patients with type 2 diabetes. Interestingly, we also found, for the first time, that total leukocyte count and its components (i.e., monocyte, neutrophil, eosinophil and basophil counts) were correlated with carotid-IMT in these patients.

Monocytes and monocyte-derived macrophages play important roles in atherosclerotic plaque progression. For example, a high monocyte count in healthy, middle-aged men was found to be predictive of coronary events [34]. In that study, the rate of coronary events was higher in subjects with a high (> 500/mm^3^) than a low (≤ 500/mm^3^) monocyte count [27], suggesting that a higher monocyte count may contribute to the state of atherosclerosis. Moreover, the baseline monocyte count was significantly higher in patients who did than did not have coronary events (365 ± 366 vs. 227 ± 197/mm^3^, p < 0.001) [34]. The monocyte count has also been reported to be an independent predictor of common carotid atherosclerosis in healthy subjects [18], and a high monocyte count (≥ 800/mm^3^) during the acute phase of acute myocardial infarction was associated with plaque progression [19]. We found that, among the leukocyte fractions, the monocyte count showed the strongest correlation with mean-CCA-IMT and max-CCA-IMT. Stepwise multiple regression analysis confirmed that the monocyte count was an independent predictor of both mean-CCA-IMT and max-CCA-IMT. Moreover, since the correlation coefficient of max-CCA-IMT was higher than that of mean-CCA-IMT, the monocyte count may be useful as a clinical predictor of diabetic macroangiopathy, especially carotid plaque, as represented by max-CCA-IMT.

Previous studies have reported that not only macrophages but also neutrophils are observed in the atherosclerotic plaque lesion [35]. Thus, it is recognized that neutrophils could be involved, at least in part, in atherosclerotic plaque progression. Although the neutrophil count was positively associated with mean-CCA-IMT and max-CCA-IMT in the present study, the correlation coefficients of these factors were lower than those of the monocyte count. Therefore, the involvement of neutrophils may be weaker than that of monocyte-macrophages in plaque formation in atherosclerosis. On the other hand, the correlation coefficients of the total leukocyte count with mean-CCA-IMT and max-CCA-IMT were also lower than those of the monocyte count. The total leukocyte count includes not only monocytes or neutrophils but also other non- or weak-associated cells, such as lymphocytes, eosinophils and basophils. Thus, the association of the total leukocyte count may be lower than that of the monocyte count. These results suggested that among the leukocyte subtypes, the monocyte count was the most useful marker for the evaluation of atherosclerosis.

Although CCA-IMT has been reported to increase with age, it never exceeded 1.1 mm in normal subjects [2,5]. Moreover, the prevalence of coronary heart disease in patients with type 2 diabetes was found to be significantly higher in patients with CCA-IMT ≥ 1.1 mm than < 1.1 mm [9]. Therefore, a CCA-IMT of 1.1 mm is usually accepted as a cut-off value for the presence of carotid atherosclerosis in patients with type 2 diabetes. We observed a positive linear correlation between monocyte count and CCA-IMT, with a monocyte count of 353/mm^3^ corresponding to a CCA-IMT of 1.1 mm in Japanese patients with type 2 diabetes.

In the present study, baPWV was not correlated with monocyte count. There are no reports of an association between baPWV and monocyte count. In general, increased IMT is thought to represent one of the earliest stages of atherosclerosis [36]. On the other hand, arterial stiffness is suggested to be a useful marker of the extent of atherosclerosis in the aorta [37]. Therefore, monocyte count may be a predictor of the early stage of atherosclerosis.

Atherosclerosis is a chronic inflammatory disease, with monocytes and monocyte-derived macrophages playing pivotal roles in the progression of atherosclerosis [10]. During this progression, the number of monocytes in blood samples may be affected by pro-inflammatory mediators, such as chemokines, cytokines and growth factors. Macrophage colony-stimulating factor (M-CSF), which is involved in the proliferation, differentiation and survival of monocytes, is increased in patients with coronary artery disease [38-40]. Thus, the association of monocyte count with CCA-IMT may involve regulation by several growth factors including M-CSF. In contrast, monocyte chemoattractant protein-1 (MCP-1) plays a critical role in the mobilization and infiltration of monocytes, and accelerates atherosclerosis in apoE-deficient mice [41]. A clinical study found that elevated plasma MCP-1 levels are associated with an increased risk of death or myocardial infarction in patients with acute coronary syndromes [42]. Moreover, MCP-1 transcript levels are higher in endothelial cells from patients with than without type 2 diabetes [43]. Therefore, overproduction of MCP-1 may contribute to the positive correlation between monocyte count and CCA-IMT. A recent study revealed that systemic insulin resistance and subclinical atherosclerosis are associated with decreased insulin receptor substrate 2 and tissue inhibitor of metalloproteinase-3 expression in circulating monocytes [44]. Therefore, the relationship between monocyte count and carotid IMT may be correlated with insulin resistance, and warrants future study.

We also found that the monocyte count in patients with type 2 diabetes was positively correlated with SBP, TG, CCA-IMT and hs-CRP, but negatively correlated with HDL cholesterol. Similar findings were observed in subjects with risk factors for CVD, in that monocyte count was positively correlated with SBP, TG, CCA-IMT and hs-CRP, and negatively correlated with HDL cholesterol [18]. Our results also showed that smoking status was correlated with monocyte count, as in the previous study [18].

Consistent with previous findings, we observed that age and duration of diabetes were positively correlated with CCA-IMT [5]. Although SBP was positively correlated with CCA-IMT, DBP was negatively correlated with CCA-IMT. Because previous studies have demonstrated that a combination of increased SBP and decreased DBP, representing a wide pulse pressure, increased carotid IMT in non-diabetic subjects [45,46], a similar phenomenon may be apparent in our study.

This study had several limitations. First, it was a single-center cross-sectional study with a relatively small number of subjects. Thus, further large-scale prospective studies are needed to confirm the association between leukocyte subtype counts and diabetic macrovascular complications. Second, there is no evidence that reducing the number of leukocytes, particularly monocytes, can prevent macrovascular complications. Indeed, treatment with HMG-CoA reductase inhibitors, which prevent the progression of atherosclerosis, significantly reduced leukocyte counts without affecting basophil counts in patients with coronary artery disease [47]. Third, the monocyte count was the only leukocyte type to be significantly and independently associated with coronary atherosclerotic progression [36]. Therefore, further studies are needed to confirm whether a reduction in the monocyte count is associated with a reduction in diabetic macrovascular complications. Finally, we did not determine the effect of pro-inflammatory mediators on the relationship between monocyte count and CCA-IMT. A recent study revealed that the inflammatory status was higher in immune cells in the carotid plaque, compared that in peripheral blood cells [48]. Further studies are needed to elucidate whether the increased monocyte count is correlated with an increase in activated monocytes in the carotid plaque, and which pro-inflammatory cytokines are involved in the increased monocyte count.

## Conclusions

To our knowledge, this study is the first to show that the numbers of leukocytes, neutrophils and monocytes were positively correlated with CCA-IMT in patients with type 2 diabetes. In particular, these results suggest that monocyte counts may be a useful predictor of diabetic macrovascular complications.

## Abbreviations

baPWV: Brachial-ankle pulse wave velocity; BMI: Body mass index; CCA: Common carotid artery; CVD: Cardiovascular disease; DBP: Diastolic blood pressure; FPG: Fasting plasma glucose; HbA1c: Glycosylated hemoglobin; HDL: High-density lipoprotein; hsCRP: High-sensitivity C-reactive protein; IMT: Intima-media thickness; JDS: Japan Diabetes Society; LDL: Low-density lipoprotein; MCP-1: Monocyte chemoattractant protein-1; M-CSF: Macrophage colony-stimulating factor; NGSP: National Glycohemoglobin Standardization Program; SBP: Systolic blood pressure; TC: Total cholesterol; TG: Triglycerides; UAE: Urinary albumin excretion.

## Competing interests

The authors declare that they have no competing interests.

## Authors’ contributions

TM designed the study; collected, analyzed and interpreted the data; and wrote the manuscript. KT, TS, NI, HK, KF, SY, DK, TK, AH, TK, SS and TN interpreted the data and revised the manuscript. HM and EA conceived and designed the study; interpreted the data and revised the manuscript. All authors approved the final version of the manuscript.
